# Direct Implementation of Intestinal Permeability Test in NMR Metabolomics for Simultaneous Biomarker Discovery—A Feasibility Study in a Preterm Piglet Model

**DOI:** 10.3390/metabo10010022

**Published:** 2020-01-01

**Authors:** Masoumeh Alinaghi, Duc Ninh Nguyen, Per Torp Sangild, Hanne Christine Bertram

**Affiliations:** 1 Department of Food Science, Aarhus University, Kirstinebjergvej 10, 5792 Aarslev, Denmark; masoumeh.alinaghi@food.au.dk; 2 Section for Comparative Pediatrics and Nutrition, Department of Veterinary and Animal Sciences, Faculty of Health and Medical Sciences, University of Copenhagen, Dyrlaegevej 68, 1860 Frederiksberg C, Denmark; dnn@sund.ku.dk (D.N.N.); pts@sund.ku.dk (P.T.S.)

**Keywords:** intestinal barrier dysfunction, leaky gut, preterm infants, lipopolysaccharide (LPS), prenatal inflammation, NMR metabolomics, lactulose

## Abstract

Measurement of intestinal permeability (IP) is often used in the examination of inflammatory gastrointestinal disorders. IP can be assessed by measurement of urinary recovery of ingested non-metabolizable lactulose (L) and mannitol (M). The present study aimed to examine how measurements of IP can be integrated in a NMR-based metabolomics approach for a simultaneous quantification of L/M ratio and biomarker exploration. For this purpose, plasma and urine samples were collected from five-day-old preterm piglets (*n* = 20) with gastrointestinal disorders (subjected to intra-amniotic lipopolysaccharide (LPS, 1 mg/fetus)) after they had been administrated a 5% lactulose and 5% mannitol solution (15 mL/kg). The collected plasma and urine samples were analyzed by ^1^H NMR-based metabolomics. Urine L/M ratio measured by ^1^H NMR spectroscopy showed high correlation with the standard measurement of the urinary recoveries by enzymatic assays (*r* = 0.93, *p* < 0.05). Partial least squares (PLS) regressions and correlation analyses between L/M ratio and NMR metabolomics data revealed that L/M ratio was positively correlated with plasma lactate, acetate and succinate levels and negatively correlated with urinary hippuric acid and glycine. In conclusion, the present study demonstrated that NMR metabolomics enables simultaneous IP testing and discovery of biomarkers associated with an impaired intestinal permeability.

## 1. Introduction

The gut is one of the most crucial organs acting as a functional barrier for interaction with the environment, and therefore, intestinal permeability (IP) plays an important role in health and disease [1]. The intestinal barrier allows the absorption of nutrients while preventing the penetration of macromolecules and microorganisms into the body [2]. Alterations in IP can be sign of an inflammatory response to pathogens [3,4].

Immaturity of the immune system and digestive tract in preterm birth may lead to the development of inflammatory diseases [2,5]. Altered IP and leaky gut have been reported during gastrointestinal complications such as necrotizing enterocolitis (NEC) [6,7] and gut-associated sepsis, suggesting failure of the immature intestine to properly develop the barrier function and adaption to extrauterine life [8,9]. NEC is the most common gastrointestinal disorder in premature infants, causing significant neurodevelopmental mortality in preterm infants [10,11]. Gut diseases are often described by a dysregulation of the colonic microbiota alongside their metabolic activities. The gut microbiome is important in the attainment of energy and nutrients in preterm neonates, which is essential for the fast development of organs together with the gut. A dysfunctional gut microbiome in preterm neonates with NEC can restrain the accessibility of symbiotic metabolites, and different neurotransmitters, which affects the development of the digestive, immune, and nervous systems in infected neonates. The immature intestinal barrier in the preterm neonate has been shown to mature postnatally. Thus, neonates with abnormal or delayed bacterial colonization of the gut may be at an increased risk of intestinal inflammation and injury due to an immature or defective intestinal barrier that allows the systemic entry of microbes, their products, or toxins from the gut lumen.

Metabolites can be considered as the signatures of biochemical activities and may be directly associated with the disease phenotypes in biological systems. Urinary metabolite levels are affected by energy and nutrient intake, metabolisms in the body and also environmental factors including microbiota that keep a close cross-talk with the intestinal system. Thus, disorders in the gastrointestinal tract might alter the metabolic levels of the body fluids which can be reflected in the blood and urine. Determination of the IP metabolite biomarkers in plasma and urine by NMR-based metabolomics could potentially be of importance in gastrointestinal disorders, particularly in preterm infants due to the fact that metabolites can reflect the excessive fermentation of undigested nutrients in the colon. In order to determine IP, conventionally a combination of non-metabolizable sugars such as lactulose (L) and mannitol (M) is implemented by oral ingestion. The ratio of the urinary excretion of lactulose, a relatively large molecule regarded as a measure of paracellular permeability, to mannitol, a small molecule regarded as a measure of transcellular permeability, is used as an indicator of the barrier function [12]. Under intestinal damage, the IP of the large molecules is increased while the permeability of the small molecules is unchanged [13]. However, in the case of severe damage, the permeability of the small molecules is decreased [14]. Therefore, L/M ratios can be considered as an indicator of IP. Calculation of the ratio of sugars has the benefit of controlling the fluctuation in urine dilution, gastric emptying, intestinal fluid volume, gastric transit time as well as impaired renal excretion, providing a direct measurement of the paracellular absorptive capacity in the gastrointestinal tract [15]. Traditionally lactulose and mannitol are determined by chromatographic methods or enzymatic assays [16,17,18]. However, ^1^H NMR spectroscopy is a rapid and non-invasive method for simultaneous detection and quantification of a large number of metabolites in complex biological fluids in a single experiment. Consequently, ^1^H NMR spectroscopy could be an appropriate approach for the simultaneous quantification of carbohydrates and exploration of metabolite biomarkers in urine [19,20]. However, with NMR spectroscopy, the number of identifiable metabolites is lower than LC-MS-based methods and is more expensive than the enzymatic assays methods.

In this study, determination of IP by using ^1^H NMR spectroscopy was first evaluated and compared with a standard measurement of urinary lactulose and mannitol by enzymatic assays. Using a piglet model for preterm infants, we hypothesized that an increased IP may be associated with perturbations in urine and blood metabolites, and metabolite biomarkers reflecting changes in IP may be revealed. To test this hypothesis, NMR metabolomics was used to evaluate the association between impaired intestinal barrier function and the metabolome of premature piglet neonates predisposed to a gastrointestinal disorder by investigating the correlations between IP measurements and the levels of plasma and urine metabolites.

## 2. Results

### 2.1. Method Elucidation

**Figure 1:**
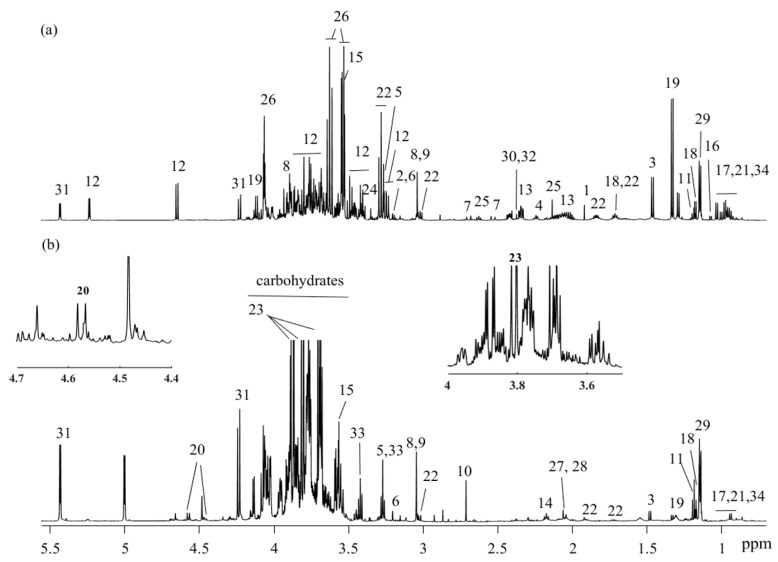
^1^H NMR spectra (obtained at 600 MHz) and assigned metabolites of (**a**) plasma and (**b**) urine. key: 1: acetate; 2: acetylcholine; 3: alanine; 4: 2-aminoadipate; 5: betaine; 6: choline; 7: citrate; 8: creatine; 9: creatinine; 10: dimethylamine; 11: ethanol; 12: glucose; 13: glutamate; 14: glutamine; 15: glycine; 16: isobutyrate; 17: isoleucine; 18: isopropanol; 19: lactate; 20: lactulose; 21: leucine; 22: lysine; 23: mannitol; 24: methanol; 25: methionine; 26: myo-inositol; 27: N-acetylglutamate; 28: N-acetylneuraminic acid; 29: propylene glycol; 30: pyruvate; 31: raffinose; 32: succinate; 33: taurine; 34: valine.

Plasma and urine samples from preterm newborn pigs were analyzed by ^1^H NMR spectroscopy, and a total of 38 and 34 metabolites were assigned in plasma and urine biofluids, respectively (Figure 1a,b).

**Figure 2:**
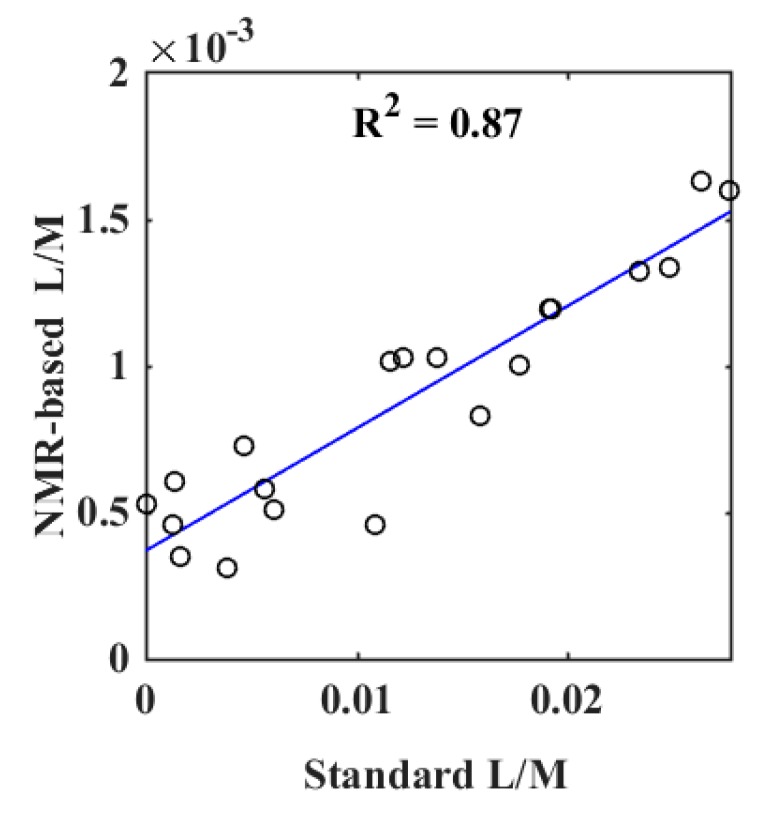
Results of a linear regression between the measurement of lactulose/mannitol (L/M) ratio by a standard assay and L/M ratio quantified by ^1^H NMR spectroscopy.

For quantification of lactulose and mannitol, NMR resonances that do not overlap with other signals should preferably be chosen. Hence, mannitol’s doublet at 3.8 ppm and lactulose’s doublet at 4.56 ppm were used for quantitative estimation of L/M ratio. The correlation between L/M ratio quantified by NMR spectroscopy and the standard measurement of L/M ratio was examined by linear regression. A strong correlation between L/M ratios determined with the standard assay and ratio obtained from quantification by NMR spectroscopy was obtained (*r* = 0.93, *p* < 0.05) (Figure 2).

### 2.2. Biomarker Exploration

**Figure 3:**
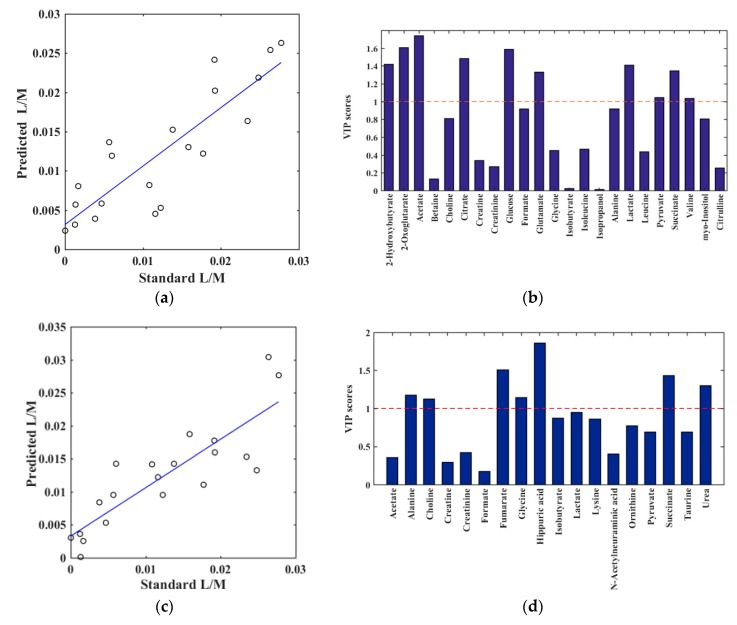
Results of partial least squares (PLS) regression models between the quantified NMR spectra (X-variables) and L/M ratio by enzymatic assay (y-variable). (**a**,**b**): Plasma metabolites and (**c**,**d**): Urine metabolites. For the plasma metabolites, the PLS model was obtained by one component, validated by leave-one-out method (R^2^ = 0.75, Q^2^ = 0.50 and root mean square error of cross validation (RMSECV) = 6.2 × 10^−3^). (**a**) plasma predicted versus quantified L/M, (**b**) plasma corresponding variable importance in projection (VIP) score plot, providing an estimation of the importance of each metabolite in PLS. A variable with a VIP score greater than 1 (dashed line) is considered important. For the urine metabolites, the PLS model was obtained by two components, validated by leave-one-out method (R^2^ = 0.73, Q^2^ = 0.51 and RMSECV = 6.2 × 10^−3^). (**c**) urine predicted versus quantified L/M, (**d**) urine VIP score plot.

In order to elucidate correlations between the L/M ratio and the metabolomes derived from NMR, partial least squares (PLS) models were performed between metabolites quantified from the obtained NMR spectra and corresponding L/M ratios. A PLS model with one component (R^2^ = 0.75, Q^2^ = 0.50, and root mean square error of cross validation (RMSECV) = 6.2 × 10^−3^) revealed that several plasma metabolites had a significant correlation with the quantified urine L/M ratio (Figure 3a and Figure S1a,b). The most prominent metabolites in the PLS regression model are illustrated by the variable importance in projection (VIP) score plot. The metabolites acetate, 2-hydroxybutyrate, 2-oxoglutarate, citrate, glucose, lactate, succinate, and glutamate all had a VIP score above 1 and can, thus, be considered important for the ability of the PLS regression model to build a quantitative relationship between the quantified metabolites and the L/M ratio (Figure 3b and Figure S1c,d). For the urine samples, a PLS regression model with two components yielded an R^2^ value of 0.73, Q^2^ value of 0.51, and RMSECV of 6.2 × 10^−3^, showing a significant correlation between the urine metabolites and L/M ratio. (Figure 3c). From the VIP score plot, it was evident that the urine metabolites hippuric acid, fumarate, succinate, urea, glycine, choline, and alanine were the most important variables (Figure 3d).

**Figure 4:**
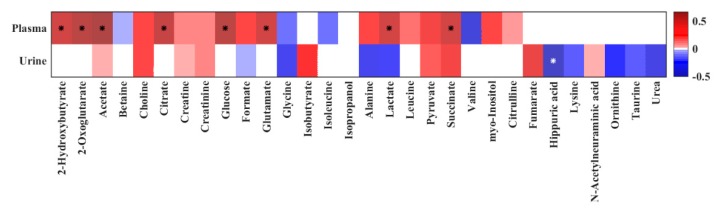
Heat map of correlation analysis between the quantified plasma and urine metabolites and quantified urine L/M ratio by ^1^H NMR spectroscopy. Stars indicate significant correlations (*p* < 0.05). The heat map is colored based on Pearson’s correlation coefficient (r). The red and blue color of every cell illustrates the Pearson’s correlation coefficient value, while deeper colors indicate higher positive (red) or negative (blue) correlation coefficients.

PLS models were supplemented with correlations analyses between plasma and urine metabolites and L/M ratio, which revealed significant positive correlations between the L/M ratio and plasma 2-hydroxybutyrate, 2-oxoglutarate, acetate, citrate, glucose, glutamate, lactate, and succinate (Figure 4). Urinary hippuric acid showed a significant negative correlation with L/M ratio. Without reaching the significance level, the urine metabolites fumarate, choline, and succinate showed positive correlation while glycine, alanine, and urea exhibited a negative correlation.

## 3. Discussion

### 3.1. Method Elucidation

Evaluation of IP is useful for the diagnosis of small intestinal diseases and evaluation of treatment intervention studies [21] especially in the early life of immature infants [5]. Minimally-absorbed and non-metabolized sugars such as lactulose and mannitol are commonly used for the assessment of barrier functions. The estimation of L/M ratios by HPLC has some analytical difficulties such as the necessity for being coupled to a pulsed amperometric, refractive index, evaporative light scattering detector, fluorescence, or mass spectrometry [16,17], which are not always convenient. Moreover, gas chromatography methods require a derivatization step [22]. Enzymatic and colorimetric methods are time-consuming and do not allow a simultaneous measurement of both sugars. ^1^H NMR spectroscopy is a rapid and convenient method without any additional sample preparation for the analysis of urinary sugars in a single analysis. In the present study, we first elucidated the potential of using ^1^H NMR spectroscopy for quantification of L/M ratio in an IP test with the ingestion of non-metabolizable lactulose (L) and mannitol (M) in preterm piglets with a gastrointestinal disorder. Correlation analysis between urinary L/M ratios determined by a standard measurement by enzymatic assays and L/M ratios determined by ^1^H NMR spectroscopy showed high correlations (r = 0.93). Thus, it was evident that significantly-correlated values to standard measurement were obtained with the applied NMR methodology, and the study demonstrated the usefulness of ^1^H NMR spectroscopy for a simple and reliable quantification of L/M ratios in urine. ^1^H NMR spectroscopy has the advantages of being rapid and non-invasive, and offers simple sample preparation. Former studies have investigated the use of ^1^H NMR spectroscopy for a simple and reliable quantification of L/M ratio in urine from healthy subjects [23] and subjects suffering from liver disease and malabsorption syndrome [20,24]. In this work, the use of a projected J-resolved NMR experiment was demonstrated to be useful for quantification of the L/M ratio [23]. To the best of our knowledge, the present study is the first to examine the use of NMR spectroscopy for quantification of L/M ratios in a model resembling infants. To combine the L/M quantification with a simultaneous metabolomics analysis, a one-dimensional nuclear Overhauser effect spectroscopy (1D NOESY) NMR experiment was applied.

### 3.2. Biomarker Exploration

Since ^1^H NMR spectroscopy is a non-selective technique that allows for the detection of numerous low-molecular weight proton-containing metabolites, the NMR-based analysis of L/M ratio enabled the simultaneous measurement of several other abundant metabolites present in urine which may reflect alterations in the gastrointestinal system. Furthermore, the NMR-based metabolomics approach was extended to also include measurements on blood to be able to explore potential blood metabolites related to IP. By employing PLS regressions and correlation analyses, the present study revealed significant correlations between IP and plasma and urine level plasma lactate, succinate, citrate, acetate, and 2-hydroxybutyrate, as well as urine hippuric acid, fumarate, and glycine. Thus, the findings suggest that perturbations in these metabolites may reflect conditions associated with intestinal barrier dysfunctions. To our knowledge, this is the first study to investigate the systemic metabolite alterations and perturbations associated with an increased IP by using a ^1^H NMR spectroscopic approach.

The present study showed that the IP has a significant positive correlation with plasma lactate. An intestinal disorder or dysbiosis may lead to an increased number of bacteria in the lumen of the infected intestine by efflux due to the impaired intestinal permeability and host defenses against bacterial overgrowth [25,26]. This bacterial proliferation could lead to an enhanced generation of bacterial metabolites and fermentation products. Lactate is the intermediate product of bacterial fermentation produced by many bacteria found in the gastrointestinal tract including *Klebsiella*, *Lactobacillus* species, *Escherichia* coli, and *Bacteroides* species [27], and are further metabolized to acetate. Succinate, in addition to other metabolites, is also produced by some bacteria as the end product of glucose fermentation. Products of bacteria metabolism can leak into the blood circulation due to an injured mucosa and increased IP in the disease process [25]. Thus, the accumulation of the bacteria fermentation products in the systemic circulation can reflect impaired gut permeability induced by some gastrointestinal disorders. Furthermore, metabolites such as lactate and succinate may also derive from the host’s own endogenous metabolism and reflect disruption of the host’s homeostasis under inflamed conditions.

In this study, an impaired IP was negatively correlated to urinary glycine. Results of some studies suggest that higher levels of glycine are linked to an improved intestinal mucosal barrier function, which is explained by a stimulation of protein synthesis and preservation of the intracellular redox states in enterocytes in piglets [28,29,30]. Furthermore, glycine concentration in the plasma of low-birth-weight neonates has been found to be lower in comparison to their normal-birth-weight counterparts [31]. Therefore, our findings corroborate other studies and suggest a possible association between a reduced glycine level in urine and intestinal dysfunction in preterm piglets.

The present study identified a negative correlation between IP and urinary levels of hippuric acid. Hippuric acid is a urinary host-bacterial co-metabolite, and its biosynthesis involves two phases, including gut microbial metabolism of aromatic dietary compounds to benzoate following conjugation of benzoate with glycine. Even though dietary benzoate can lead to differences in the urine hippuric acid levels [32], its lower level is reported in inflammatory bowel diseases and Crohn’s disease due to an altered gut microbial metabolism [33,34]. Alterations in the urine level of hippuric acid may thus reflect systemic effects of an altered gut microbial metabolism.

## 4. Materials and Methods

### 4.1. Animal Experimental Procedures

All animal experiments were approved by the Danish National Committee of Animal Experimentation. To investigate the IP and metabolic influences of prenatal lipopolysaccharide (LPS), five pregnant sows (Large White × Danish Landrace × Duroc) were operated by laparotomy at d 103 of gestation (term at 117 ± 2 days of gestation), and every fetus received an IA dose of 1 mg LPS (from E.coli 055:B5, Sigma Aldrich, Copenhagen, Denmark) in an area near to the mouth, as previously described [24]. Afterward, cesarean section was performed to deliver the preterm pigs at d 106 (89–92% gestation age). A detailed description of the animal procedure and nutritional composition can be found elsewhere [35,36,37]. Clinical circumstances and fecal features were assessed twice daily as previously described [24]. The animal procedures were all approved by the Danish National Committee on Animal Experimentation (2014-15-0201-00418).

### 4.2. Intestinal Permeability Test

The IP was evaluated by providing an enteral bolus of 15 mL/kg of a 5% lactulose (Sigma-Aldrich, Copenhagen, Denmark) and 5% mannitol solution (Sigma-Aldrich, Copenhagen, Denmark) exactly 3 h before euthanasia. Urine was collected at the time of euthanasia and stored at −20 °C until assayed. Concentrations of lactulose and mannitol were measured in urine by an enzymatic spectrophotometric method (Pentra 400, Irvine, CA, USA). In the presence of mannitol dehydrogenase, mannitol was oxidized by NAD into the fructose and NADH. The amount of NADH was detected by spectrophotometry at 340 nm. Lactulose was also hydrolyzed into galactose and fructose. Fructose was then catalyzed into fructose-6-phosphate and to glucose-6-phosphate by phosphoglucoisomerase. Glucose-6-phosphate was dehydrogenated by adding glucose-6-phosphate-dehydrogenase in the presence of NADP to form NADPH. The concentration of NADPH is proportional to the concentration of lactulose and can be measured spectrophotometrically at 340 nm. L/M ratios quantified by enzymatic assay are provided in the Supplementary Materials.

### 4.3. NMR Sample Preparation

Amicon Ultra 0.5-mL 3 kDa (Millipore, Billerica, MA, USA) was used to filter the thawed plasma samples at 10,000× *g* at 4 °C for 3 h to remove lipid and proteins. For NMR measurement, 300 µL plasma sample, 250 µL D_2_O and 50 µL D_2_O containing 0.05% sodium trimethylsilyl-[2,2,3,3-2H4]-1-propionate (TSP; Sigma-Aldrich, St. Louis, MO, USA) as an internal chemical shift reference was added to a 5 mm NMR tube (Wilmad, Vineland, NJ, USA). For the urine samples, 100 µL phosphate buffer in D_2_O [38] (pH = 7.4) containing 0.05% TSP was transferred to 500 µL of thawed urine in a 5 mm NMR tube.

### 4.4. NMR Data Acquisition and Preprocessing

NMR spectra were recorded on a Bruker Avance III 600 MHz NMR spectrometer (Bruker BioSpin Gmbh, Rheinstettten, Germany) equipped with a 5-mm ^1^H TXI probe and operating at a ^1^H frequency of 600.13 MHz. A target temperature of 298 K and a relaxation delay of 5 s were applied. A total of 128 free induction decays (FIDs) were acquired, and the acquisition parameters included 32,768 data points, a spectral width of 7288 Hz and an acquisition time of 2.25 s. A one-dimensional nuclear Overhauser effect spectroscopy (1D NOESY) experiment with a single 90° pulse sequence and pre-saturation of the water resonance was conducted.

The spectra were processed with zero-filling prior to Fourier transformation. All spectra were referenced to the TSP signal at 0.0 ppm. An experimental window function with a line-broadening factor of 0.3 Hz was applied to all FIDs before Fourier transformation. The resulting spectra were manually phase- and baseline-corrected by polynomial using the Topspin^TM^ 3.0 software (Bruker BioSpin, Gmbh, Rheinstetten, Germany). The NMR resonances were assigned based on 2D NMR spectroscopy, the Human Metabolome Database [39], existing literature [40,41,42] and Chenomx NMR Suite 7.7 (Chenomx Inc, Edmonton, AB, Canada).

### 4.5. Data Analysis

Quantitative analysis of metabolites was performed on data by the integration of peak areas using TopspinTM 3.0 software (Bruker BioSpin, Gmbh, Rheinstetten, Germany). The metabolites were quantified relative to the area under the TSP peak. The analysis was performed on quantified NMR metabolites obtained from plasma and urine samples of the same piglets. The final NMR data sets of plasma and urine had the size of 20 × 23 and 20 × 18, respectively, with the rows representing the quantified metabolites of each piglet and the columns representing each of the quantified metabolites.

Linear regression was performed between the standard measurement of urinary L/M and the quantified urine L/M ratio by NMR spectroscopy. To determine whether the IP was correlated with the quantified metabolites of plasma and urine profiles, partial least squares (PLS) regressions were performed between the quantified metabolites (X) and quantified urine L/M ratio by enzymatic assay (y). Orthogonal signal correction (OSC) [43] was performed on the X matrix and the data were autoscaled prior to PLS regressions. The quality of the model was evaluated by the goodness-of-fit parameter R^2^ and the predictive ability parameter Q^2^ using the leave-one-out validation. Variable importance in projection (VIP) scores were calculated to evaluate the importance of each metabolite in the projection used in a PLS model [44]. The metabolites with VIP scores greater than 1.0 were regarded as important variables.

Moreover, Pearson’s correlations between quantified plasma and urine metabolites and the urinary L/M ratio quantified by NMR spectroscopy were calculated and coefficients and *p*-values of correlations were evaluated. The concentration of the quantified metabolites as well as the L/M ratios quantified by NMR spectroscopy are provided in the Supplementary Materials. Matlab (version R2016b, MathWorks Inc., Middlesex, MA, USA) was used for data analysis and visualization.

## 5. Conclusions

NMR-based metabolomics analysis of LPS-induced preterm piglets revealed that ^1^H -NMR spectroscopy is a reliable and rapid technique to estimate the L/M ratio in urine samples. To our knowledge, this is the first study that has combined NMR-based L/M determination with NMR-based metabolomics to assess IP biomarkers in preterm piglets with systemic inflammation. Our findings suggest that increased plasma lactate, acetate, and succinate levels, as well as decreased levels of urinary hippuric acid and glycine can be considered potential markers of an impaired IP induced by gastrointestinal disorders.

## Supplementary Materials

The following are available online at https://www.mdpi.com/2218-1989/10/1/22/s1, Figure S1: Leave-one-out cross validation for PLS model (a) and (b) plasma NMR data, (c) and (d) urine NMR data. (a) Q^2^ value for PLS on plasma data, and (b) RMSECV for PLS model on plasma data. (a) Q^2^ value for PLS on urine data, and (b) RMSECV for PLS model on urine data.
