# Supplementary material for: Direct Implementation of Intestinal Permeability Test in NMR Metabolomics for Simultaneous Biomarker Discovery—A Feasibility Study in a Preterm Piglet Model
Source: Metabolites. 2020 Jan 1;10(1):22. doi: 10.3390/metabo10010022 (PMC7022985; doi:10.3390/metabo10010022)
Supplement: Supplementary file 1 [file metabolites-10-00022-s001.pdf]

Supplementary materials:

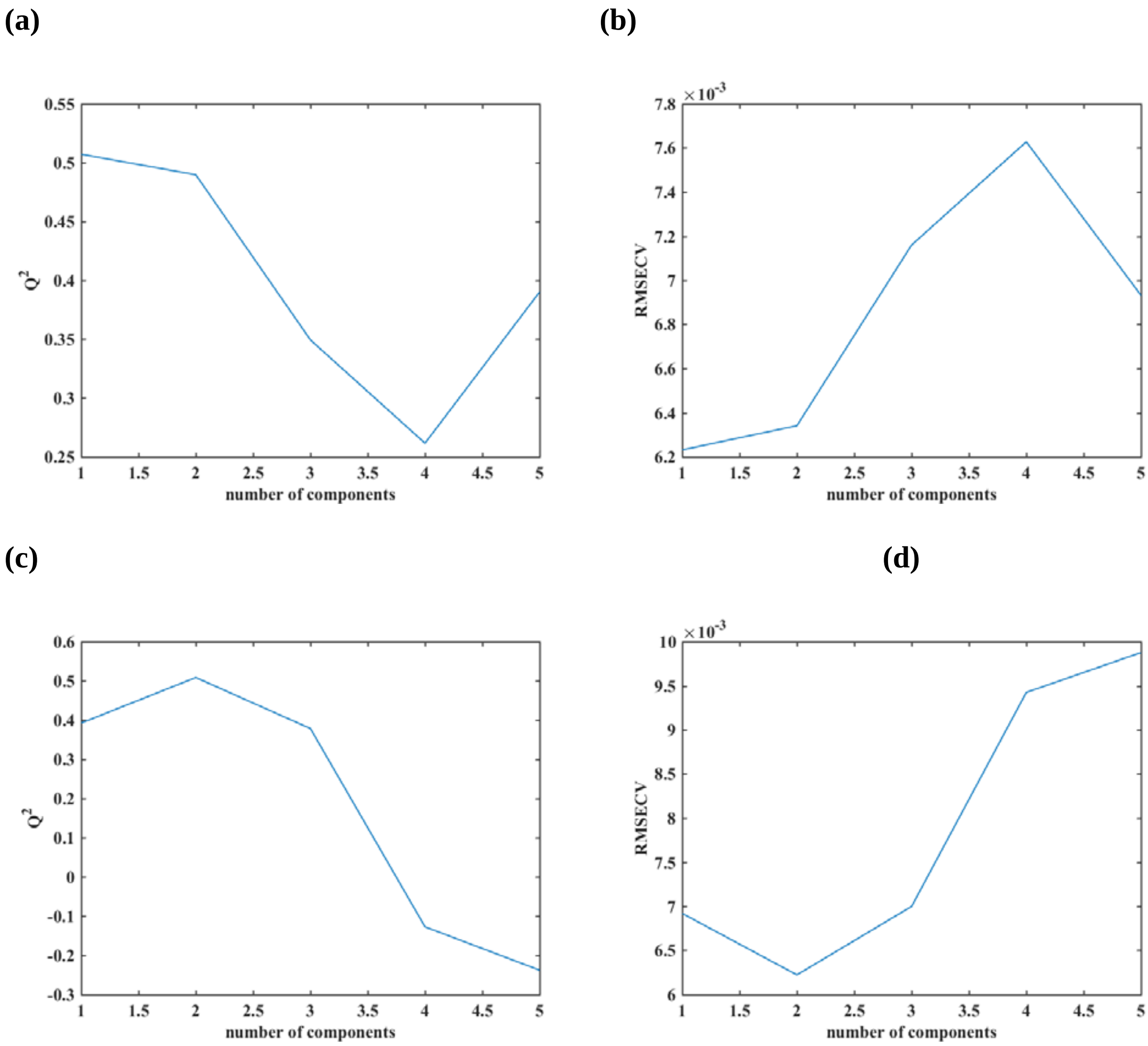

Figure S1. Leave-one-out cross validation for PLS model (a) and (b) plasma NMR data, (c) and (d) urine NMR data. (a) Q^2 value for PLS on plasma data, and (b) RMSECV for PLS model on plasma data. (a) Q^2 value for PLS on urine data, and (b) RMSECV for PLS model on urine data.
